# Enhancing national fitness legislation in the context of promoting a healthy China

**DOI:** 10.3389/fspor.2025.1598339

**Published:** 2025-07-07

**Authors:** Quansheng Wang, Guoqing Han, Lansong Huang

**Affiliations:** Discipline Inspection and Supervision School, Shandong University, Weihai, China

**Keywords:** healthy China, national fitness, legislation, smart sports, sports law of the People's Republic of China

## Abstract

The Central Committee of the Communist Party of China and the State Council issued the *“*Healthy China 2030” planning outline as a program of action to promote a healthy China in October 2016. The document emphasizes the importance of national fitness in improving the country's health and recognizes legislation on national fitness as one of the strategic goals to achieve a healthy China. In this context, improving China's national fitness legislation is of significant value in promoting the development of a healthy China and enforcing sports laws. Based on the existing issues in the current national fitness legislation, China should enhance the system of sports rights for citizens and improve citizens’ health by incorporating a protection clause for sports rights into the National Fitness Regulations and the revised sports law. This can be achieved by restructuring the relationship between citizen sports rights and government responsibilities and by exploring various dispute resolution mechanisms.

## 1 Introduction

The 13th National Five-Year Plan, in March 2016, integrated the national fitness strategy and the Healthy China strategy, highlighting that national fitness is a crucial element in building a healthy China and adding new importance to sports development in China (1, 2). In October of the same year, the Central Committee of the Communist Party of China and the State Council issued the 2030 Healthy China Plan as a program to develop a healthier China. National fitness is vital to this effort, and implementing national fitness legislation is a strategic goal (3). Against this backdrop, the improvement of national fitness legislation in China is of great significance in promoting the construction of a healthy China and the rule of law in sports (4).

Research on sports law has developed alongside an increase in sports legislation in various countries. It has undergone the process of evolving into both public and private law, resulting in the intersection of public and private law (5–7). The National Fitness Act is a branch of sports law that focuses on safeguarding national sports and public service through legislation (8, 9). Currently, academic research of relevance focuses on the following three aspects.

First, research on sports rights of citizens, in light of the growing awareness of civil rights, indicates that sports rights are increasingly recognized as an integral part of human rights on an international scale (10, 11). Some scholars pointed out in their discussion of the development of human rights that early concepts of human rights were not inclusive, as they excluded women, the working class, and non-European ethnic groups. They also noted that the early rights to sport were not inclusive. By examining cases of antiracism, the promotion of gender equality, and the protection of athlete rights, two scholars argued that sports rights should embody the principles of equal rights and substantive equality. They also emphasize that sports rights should prioritize achieving substantive equality (12, 13). In the realm of rights to sports by marginalized groups, international academic research has focused primarily on the rights of women and individuals with disabilities. In terms of the development of women's sports rights, Sweden, Finland, Norway, New Zealand, the United Kingdom, Australia, and Canada have made significant progress toward gender equality in sports participation. They have developed various frameworks and policies for gender equality to provide women with the opportunity to participate in the sport of their choice. Based on these findings, Burton, an American scholar, suggested that, although female involvement in sports has grown, women remain underrepresented in leadership roles at all levels of athletics (14). This phenomenon remains unchanged. Danish scholars Adam B. Evans and Gertrud U. Pfister state that women are underrepresented in all regions and sports worldwide. This impedes the achievement of women's sports rights (15). In terms of the development of sports rights for people with disabilities, Canadian scholars recommend that countries adopt policies or legislation to promote sports and physical activity for people with disabilities and to ensure the realization of the substantive equality of sports rights for people with disabilities (16).

Second, the research focused on national fitness policies (17–19). National fitness-related policies are a strategy developed countries such as Europe and North America have adopted and paid attention to. British scholars Kohl, Craig, Lambert, and others believe that the national fitness policy should promote close cooperation between the sports sector and other departments, such as health. They advocate for collaboration among government, nongovernmental organizations, and individuals, rather than relying solely on government implementation of the policy (20). Some scholars conducted a comprehensive evaluation and systematic analysis of 130 sports policies in Europe. In terms of policy concepts, they believe that sports policies should prioritize “national fitness” and promote the deep integration of health and fitness (21). In terms of policy objectives, it is proposed that specific and quantifiable indicators should be set during policy formulation. It is best to adopt the SMART method, which means that the objectives should be specific, measurable, achievable, relevant, and have a clear deadline (22).

Third, research on the legal framework for national fitness in China (23, 24). China's National Fitness Regulations, introduced in 2009, are the country's first specialized regulations aimed at developing and safeguarding national fitness. Some scholars have proposed that the National Fitness Regulations are institutional arrangements designed to promote public sports services (25). Since then, “national fitness” has no longer been limited to the concept of mass sports in the outline of the national fitness plan. It now includes sports public service and sports market management, reflecting the state's commitment to safeguarding citizens' fitness rights. Some scholars believe that the current supply of public sports facilities in China is insufficient and argue that legislative protection is also inadequate (26). Some scholars agree that the current supply of rural sports resources is insufficient and structurally imbalanced (27). To achieve this goal, some scholars have proposed enhancing sports law enforcement and advancing sports administrative accountability (28). Some scholars have suggested the introduction of a multi-subject competition mechanism and a market-oriented operation mechanism (29). Some scholars proposed building a government performance evaluation system with public satisfaction as the core to promote the transformation of the government into a “service-oriented government” (22).

Based on existing research findings, this study explores the historical evolution of national fitness legislation in China and the current status of national fitness legislation in contemporary China. Based on an analysis of defects and deficiencies in contemporary national fitness legislation in China from the perspective of promoting a healthy China, this study proposes suggestions and measures to improve national fitness legislation with a health-oriented approach.

## 2 Materials and methods

This paper selects China's current effective legislation on mass fitness as its analytical material, covering the period from the founding of the People's Republic of China to the present day, i.e., from 1949 to the present. Regarding the entities involved in national fitness legislation, under China's current legislative system, these range from the highest legislative body—the National People's Congress and its Standing Committee- to the lowest-level legislative bodies, the people's governments of cities divided into districts. The scope of legal normative documents includes laws, administrative regulations, local regulations, autonomous region regulations, single-issue regulations, and rules.

The research methods used in this article include, first, historical research methods. This article uses a historical perspective to analyze the development of China's national fitness legislation, examining changes in the form and content of such legislation from a historical angle to grasp the distinctive features of China's national fitness development. Second, a combination of normative and empirical analysis methods is used. Normative analysis, which is also descriptive analysis, involves content analysis of current legal norms to examine the main content of China's mass fitness legislation and its social impact; the empirical analysis primarily examines the actual effects of current mass fitness legislation, primarily through data to reflect the promotion and development of China's mass fitness movement.

## 3 Development of national fitness legislation in China

The origin and development of the global fitness movement is a process that transcends time and space, integrating social change and the evolution of health concepts. The core lies in the continued pursuit of health and quality of life by humanity (30, 31). The origins of the mass fitness movement can be traced back to ancient times, such as the ancient Greek Olympics and the ancient Chinese martial arts (32). Although the modern concept of mass fitness had not yet been developed at that time, the basic elements of sports activities already existed. The wave of the industrial revolution of the nineteenth century profoundly changed the way humans lived and worked. Mechanized production led to a reduction in physical labor, and urbanization accelerated population concentration, but public health conditions lagged, infectious diseases raged, and workers' health problems became prominent. Against this backdrop, Germany took the lead in passing the Gymnastics Act, incorporating gymnastics into the national education system and emphasizing the importance of collective exercise in improving physical fitness. Meanwhile, Sweden developed a fitness system centered on medical gymnastics, which combines exercise with disease prevention. These early practices laid the institutional foundation and scientific concepts for national fitness (33). At the same time, the revival of the modern Olympic movement (1896) further promoted the popularization of sports. Pierre de Coubertin proposed the concept of “sports equals peace” and emphasized the importance of sports for individual physical and mental development. The Olympic Games became a symbol of the global spirit of sports and inspired countries around the world to attach importance to mass sports (34).

Since the founding of the People's Republic of China in 1949, the national fitness legislation of China has undergone a gradual establishment and development process.

### 3.1 The embryonic stage of legislation (1949–1978)

The development of national fitness legislation began as early as the socialist construction period (1949–1978) (31). First, the Chinese Constitution provides explicit provisions. At the beginning of the establishment of the People's Republic of China, Article 48 of the Common Program of the Chinese People's Political Consultative Conference (1949) explicitly outlined Chinese citizens, highlighting the country's significant emphasis on sports. Article 94(2) of the 1954 Constitution later replaced “sports” with “physical strength.” During that time, the right to participate in sports was considered part of the right to education. The 1954 Constitution of China restricted sports rights to “youth” rather than extending them to all citizens. Subsequently, Article 12 of the 1975 Constitution redefined the term “sports”, but sports rights were not listed separately. Instead, they were listed alongside education, culture, scientific research, and health, and the article only stated that sports should serve the proletariat, without specifying their development and importance. Articles 13 and 52 of the 1978 Constitution highlight the significance of sports.

Second, in October 1958, the former State Sports Commission issued the Regulations on the Physical Education System for National Defense through Labor. The regulations aimed to “encourage people to actively participate in physical activity.”

Third, China promulgated the “National Physical Exercise Standards Regulations” and simultaneously implemented the broadcast gymnastics system in April 1975.

The aforementioned legal documents demonstrate that China places great importance on physical exercise (4).

### 3.2 Recovery and reconstruction phase (1978–1993)

The period from 1978 to 1995 marked the restoration and reconstruction stage of national fitness legislation. At the end of 1978, the Third Plenary Session of the Eleventh Central Committee of the Party proposed a basic policy of “developing socialist democracy and improving the socialist legal system.” In addition, Article 33, paragraph 3 of the 1982 Constitution clearly states that “the state respects and protects human rights.” The right to participate in sports is not explicitly stated. However, this is one of the fundamental implications of human rights. Therefore, the Constitution of China protects the right to sports. In March 1990, the State Council promulgated the Regulations on School Physical Education Work. It has made adequate provisions for extracurricular physical education training, physical education teachers, and sports venues. This improvement improved the status of physical education in schools. This has laid the foundation for the opening of sports facilities at national fitness schools to the public in this new era. In addition, in August 1982, the State Sports Administrative Department issued the “National Physical Exercise Standards” to replace the “National Physical Exercise Standards Regulations.” In April 1986, the former State Physical Culture and Sports Commission issued the “Decision on the Reform of the Sports System (Draft)” (35). The draft proposes 53 specific measures to reform the sports system, one of which is to develop a sports law that aligns with the current development situation in China.

### 3.3 Rapid development stage (1993–2009)

From 1993 to 2009, national fitness legislation underwent rapid development. At the beginning of 1993, the directors of the National Sports Commission proposed describing an outline of the National Fitness Plan to improve the nation's physical fitness. In December 1993, the former National Physical Culture and Sports Commission issued the “Social Sports Instructor Technical Hierarchy System.” Social sports instructors are volunteers dedicated to promoting physical fitness at the national level. The promulgation of the law is a response to the initiative outlined in the “National Fitness Program Outline.” This designation also marks the official establishment of China's social sports instructor system (36). In June 1995, the State Council issued an outline of the National Fitness Program. The outline is of great strategic significance (37). In August 1995, the “Sports Law” was promulgated, and Article 11 clearly states that a national fitness program was implemented. The “Sports Law” is the foundational legislation that governs sports in China, marking the official beginning of sports governance in the country. The national fitness legislation is one of the integral components of the sports rule of law in China. The development of national fitness legislation is closely intertwined with sports legislation, and the enactment of sports laws has provided a strong foundation for the significant progress of national fitness legislation in China (38). In 1996, the State Sports Commission held the first national sports legal work conference, marking the first time the commission had publicly articulated the goal of establishing the China Sports Law Framework. In September 1998, the promulgation of the Interim Measures for the Management of the Sports Lottery Public Welfare Fund provided a legislative guarantee for the funding source of national fitness initiatives in China. Since then, the department has enacted regulations such as the Fitness Instructor Technical Rating System (1998), Roller Skating Activities Management Measures (1999), and National Sports Venue Maintenance Special Subsidy Funds Management Measures (1999). The implementation of various measures has contributed significantly to the development of national fitness legislation. “These measures include the National Sports Venue Maintenance Special Subsidy Funds Management Measures of 1999, the Sports Private Non-Enterprise Units Registration Review and Management Interim Measures of 2001, the Rural Sports Work Interim Provisions of 2002, the Domestic Mountaineering Management Measures of 2003, the Public Cultural and Sports Facilities Regulations of 2003, and the Fitness Qigong Management Measures of 2006” (39).

### 3.4 High-quality development stage (2009–present)

In August 2009, the introduction of the “National Fitness Regulations” marked the beginning of high-quality development in national fitness legislation in China. The law clarifies the responsibilities of various entities, including the government, enterprises, schools, and social groups. It also provides special protection to vulnerable groups, such as individuals with disabilities, older adults, and rural residents. This legislation represents the first comprehensive national fitness law within a unique context in China. It is also a milestone for national fitness in terms of standardization, the rule of law, democracy, and science. At the same time, the national fitness legislation has completed the historic transition from single legislation to comprehensive legislation (29).

In October 2011, the State General Administration of Sport promulgated the Measures for the Management of Social Sports Instructors. The term “social sports instructors” in this law refers to a group of instructors who work for the public welfare and do not receive remuneration. This concept inherits the public-benefit nature of instructors in the Technical Hierarchy of Social Sports Instructors (1993). The “Social Sports Instructor Management Measures” are the legislative outcome of the comprehensive revision of the “Social Sports Instructor Technical Hierarchy System (1993).” This revision further enhances the voluntary service system in China's national fitness legislation. In February 2013, the State General Administration of Sport issued the “Management Measures for the Licensing of High-Risk Sports.” The term “high-risk sports” was initially introduced in the “Regulations on National Fitness” as the focal point of this legislation. These regulations outline the requirements for conducting high-risk sports, specify the entity responsible for the administrative license, detail the procedural aspects, and define the extent of their application. The “Management Measures for the Licensing of High-Risk Sports Projects” have been further standardized and made more specific compared to the original document. This ensures that “the application of standardization in the supervision of high-risk sports activities has been incorporated into the legal framework” (40). In October 2014, the State Council promoted national fitness as a national strategy. They discussed this issue in a document titled “Several Opinions on Accelerating the Development of the Sports Industry and Promoting Sports Consumption.” This implies that national fitness has a new strategic position in the development of sports excellence. National fitness not only can enhance the physical well-being of a nation but also can drive the transformation of the consumption patterns of the sports industry. Becoming an important source of power can stimulate domestic demand. In October 2015, at the Fifth Plenary Session of the 18th CPC Central Committee, the construction of a healthy China was officially identified as a national strategy. In October 2016, the Central Committee of the Communist Party of China and the State Council issued an action plan to build a healthy China, known as the “Healthy China 2030” Plan Outline. The State Council issued the School Sports Work Regulations in March 2017. In January 2020, the “Measures for the Management of Sports Events” were issued. This legislative response and norm were established to support the national fitness initiative, encouraging society to organize a variety of national fitness events to promote the construction of a healthy China (41).

In June 2022, the revised “Sports Law” was published to the public, marking the second amendment since the promulgation of the “Sports Law” in 1995. One of the five new initiatives is the nationwide implementation of the national fitness strategy. The original second chapter, “Social Sports,” was changed to “National Fitness,” and the existing provisions of the chapter were comprehensively and fully revised. This move is significant for national fitness legislation. For a long time, national fitness legislation has been centered around the “Sports Law.” However, the specific content related to the promotion of national fitness is limited. After the amendment, the “National Fitness Regulations” have legal standing as administrative regulations. The revision of the “Sports Law” has enhanced the national fitness legislation by establishing a comprehensive system that integrates the “Sports Law,” supported by the “National Fitness Regulations” and “School Sports Work Regulations,” and guided by departmental sports regulations. The further development and enhancement of the system is based on local fitness regulations and policies issued by local authorities (42, 43).

## 4 Current status of national fitness legislation in China

As of March 13, 2023, China had 27 central national fitness laws, including 1 law, 7 administrative regulations, and 19 departmental rules, accounting for 3.70%, 25.93%, and 70.37% of the total, respectively. This is in addition to thousands of normative documents (Table 1).

**Table 1:** List of national fitness legislations.

No	Name	Date of publication
1	Sports law of the People's Republic of China	August 1995
2	National standard regulations on physical exercise	April 1975
3	“National physical exercise standards”	July 1982
4	Measures for the implementation of national physical exercise standards	December 1989
5	Regulations on School Physical Education	March 1990
6	Public cultural and sports facilities ordinance	June 2003
7	Lottery regulations	May 2009
8	National fitness regulations	August 2009
9	Regulations of the state sports commission on awarding sports science and technology progress	September 1989
10	“Technical hierarchy of social sports instructors”	December 1993
11	Measures for archival management of large-scale sports games	October 1996
12	The technical hierarchy of fitness instructors	January 1998
13	Interim provisions for the administration of national water sports business activities	September 1998
14	Interim provisions for the administration of national water sports business activities	September 1998
15	Measures for the administration of special subsidies for the maintenance of national sports venues	November 1999
16	Administrative measures for roller skating activities	August 1999
17	Administrative measures of the general administration of sports of the state on swimming activities in open water (trial)	January 2000
18	Disciplinary regulations for staff of national comprehensive games	May 2000
19	Interim measures for the administration of fitness qigong	September 2000
20	Interim measures for examination and management of registration of private non-enterprise sports units	January 2001
21	Measures for the administration of mountaineering in China	July 2003
22	Health qigong management measures	November 2006
23	Measures to control firearms in competitive shooting sports	August 2010
24	Measures for the administration of social sports instructors	October 2011
25	Rules for the implementation of regulations on lottery management	January 2012
26	Administrative measures for licensing high-risk sports	February 2013
27	Administrative measures for sports events	January 2020

The central national fitness legislation covers 11 main categories: comprehensive sports regulations, fitness regulations, fitness market and management, fitness venues and facilities, instructors, sports events, funds, physical fitness monitoring, school sports, high-risk activities, and leisure and fitness activities. For the inclusion of special interest groups such as rural sports enthusiasts, older people, disabled people, and traditional ethnic items in project activities, only normative documents are available for legislation. These documents have limited normative effectiveness and are scarce in quantity. Legislation in these three categories is lacking compared to other categories.

China's national fitness legislation has several characteristics. First, it includes various laws and regulations. There is only one effective law and four administrative regulations. These are not only a few but also include the “Lottery Management Regulations,” which are not specific national fitness-security legislation. Second, the level of legislation is low. Departmental regulations account for 70.37% of the total national fitness legislation, and there are thousands of normative documents. Some important aspects of national fitness, such as regulations for special groups, are only specified in departmental normative documents. Third, the number of national laws supporting fitness is limited. There are only three pieces of legislation based on the National Fitness Regulations: measures for the management of social sports instructors, licensing of high-risk sports projects, and sports event management.

## 5 Defects and deficits in China's national fitness legislation

The national fitness legislation provides a legal guarantee for developing national fitness initiatives in our country. Despite various constraints, there are still numerous issues with our national fitness legislation. The presence of these issues hinders the realization of sports rights for our citizens and fails to meet the growing demand for physical fitness among the population.

### 5.1 Misalignment between government responsibilities and sports rights

China's national fitness legislation is generally enforced to regulate fitness standards and promote the advancement of physical fitness (44). The National Fitness Regulations and the supporting legislation for local fitness laws do not adequately consider the orientation and purpose of the legislative value in the development of the concept of right-based fitness. Administrative departments, especially sports authorities, dominate the space in almost all national fitness laws, highlighting the administrative nature of the national fitness legislation system. The emphasis on the authority of administrative departments in legal documents will inevitably lead to unintentional omissions in protecting the rights and interests of sportsmen and women in general planning. The misuse of power can also lead to violations of citizens' sports rights. The imbalance of rights and power structures has led to a lack of internal motivation and autonomy in the development of the sports industry in our country, resulting in excessive dependence on state support.

### 5.2 Sports rights are not equally protected

Based on the theory of substantive equality, sports rights, as the institutionalized distribution mechanism of sports resources, should provide special protection to vulnerable groups (45). Following the introduction of the National Fitness Strategy and the Healthy China Action, there has been a greater social emphasis on ensuring the rights of vulnerable groups to sport. In the context of comprehensively building a moderately prosperous society and achieving a comprehensive victory against poverty, there is a growing awareness of the importance of providing special protection for vulnerable groups. This involves ensuring an equal distribution of social wealth and welfare.

First, the sports rights of rural residents have been neglected. The National Bureau of Statistics published the seventh Bulletin of the National Census. This indicates that 36.11% of China's total population lives in rural areas. Despite being a significant demographic, the revised sports law overlooks this group (46).

Second, the protection of women's sports rights is also inadequate. Under the comprehensive influence of feminist theory, international sports culture, and other factors, Chinese women's sports rights have made significant progress. On the one hand, China has never introduced legislation or policies specifically addressing women's sports and the protection of women's sports rights. There are no provisions related to “sports” or “fitness” in the Law on the Protection of Women's Rights and Interests, which is specialized legislation aimed at safeguarding women's rights. However, the Sports Law, which was recently revised, is the only national fitness legislation that specifically aims to achieve substantive equality in women's sports rights. On the contrary, the National Fitness Regulations and local legislation only address equality of rights and qualifications (47).

Third, protection of sports rights for older people, disabled people, ethnic minorities, and other vulnerable populations is inadequate (48). The Law on the Protection of the Rights and Interests of the Elderly, the Law on the Protection of the Disabled, and the Law on Regional Ethnic Autonomy all ensure the realization of the sports rights of the respective groups. However, in national fitness legislation, the sports rights of these special populations are overlooked to varying degrees, leading to inconsistent laws and regulations to protect these groups. They hinder the realization of the sports rights of these special groups.

### 5.3 Structural shortage of fitness resource allocation and supply capacity

The realization of citizen sports rights cannot be separated from the provision of fitness resources, such as sports venues, facilities, and funding. The demand for fitness resources among citizens is also increasing with the development of the economy. However, the reality is that sports funding is limited, and in some regions it is not even included in the government budget (49). Under the impact of the pandemic, the expenditure budget of the State General Administration of Sport was significantly reduced compared to the pre-epidemic period due to slow progress and insufficient budget implementation. The carryover of special sports funds occurs frequently and is sometimes embezzled, which impacts the efficiency of fund use. Some regions are struggling with the challenge of limited land for fitness centers and other facilities. Fitness facilities are struggling with aging infrastructure, rust, and significant damage, all of which pose security risks. The current material situation is unable to meet the needs of citizens, leading to a structural shortage in the allocation and supply capacity of fitness resources, making it challenging to realize the rights of the citizens to sports (50).

### 5.4 The sports relief system is not perfect

The realization of sports rights cannot be separated from support. From a necessity point of view, a diversified dispute resolution mechanism for sports rights is essential and inevitable. The comprehensive dispute resolution mechanism should include reconciliation, mediation, litigation, arbitration, state compensation, administrative rulings, and reconsideration to accommodate the diverse demand channels for sports rights relief among citizens. At present, there are four main types of sports rights disputes: disputes related to events, athletes, and coaches' contracts, namely, contract sports disputes (51). Disobedience in sports management can lead to conflicts. These disputes are called management sports disputes. Disputes caused by dissatisfaction with technical penalties in sports events are known as technical sports disputes, whereas disputes arising from citizens or special groups who fail to realize their guaranteed legal sports rights are referred to as legal sports disputes. From the perspective of the revised sports law, although it has added a chapter on sports arbitration, this chapter is only applicable to specific types of sports disputes, mostly those managed by sports organizations, and does not apply to other types, especially the sports rights relief of ordinary citizens. Currently, disputes about protective sports are resolved mainly through legal action. However, some scholars have pointed out that current national fitness legislation often lacks robust judicial enforceability, making it unreliable for the resolution of sports disputes (52).

## 6 Measures to improve national fitness legislation within the framework of healthy China

### 6.1 Add a sports rights protection clause to the national fitness regulations

With the latest revision of the “Sports Law,” the sports rights of vulnerable groups have been guaranteed to some extent. The “National Fitness Regulations,” as a subordinate law of the “Sports Law,” should be consistent with the “Sports Law” and undergo timely improvements and amendments to establish coherence between the laws (35).

First, the scope of protection for vulnerable groups should be expanded. Article 5 of the revised “Sports Law” classifies minors, women, older individuals, and people with disabilities as vulnerable groups. Therefore, the “Regulations on National Fitness” should be amended to reflect this classification. At the same time, it is important to consider that China's urban-rural dual structure places rural residents as the primary vulnerable group, especially migrant workers who move between urban and rural areas in a transitional “vacuum zone” (53). Ethnic minorities are a group protected by the Constitution. However, sports law lacks this provision. It may be included after Article 4, paragraph 1, of the National Fitness Regulations: “The State provides special protection for the rights of minors, women, older individuals, the disabled, ethnic minorities, rural residents, etc., to engage in national fitness activities.” Relevant provisions to safeguard sports rights of vulnerable groups, including women, older people, and disabled people, can be incorporated into laws on the protection of rights and interests of women, the protection of rights and interests of older people, and the protection of the disabled. This measure will establish coordination and alignment with national fitness regulations. Second, the Regulations on National Fitness and local legislation should strengthen and refine the protection provisions outlined in the Sports Law. Additionally, in the chapter on “National Fitness Activities,” provisions should be included to promote equitable access to public sports facilities for minors, women, older individuals, individuals with disabilities, ethnic minorities, and rural residents. To protect women's sports rights, it is recommended to include specific protection provisions for women's sports rights in the National Fitness Regulations, along with the development of policies and regulations tailored specifically for women's sports (54). The equal protection of women's sports rights and qualifications will be transformed into substantive equality protection.

### 6.2 Reconstructing the relationship between citizen sports rights and government duties

There is a natural opposition between citizen sports rights and government duties determined by the economic foundation. Currently, the government's power clause in the national fitness legislation outweighs the right clause in both quantity and intensity, resulting in an imbalanced “power-right” structure that affects the maximization of sports interests. Therefore, it is necessary to achieve a balance between “power and right” through legislation (55). This balance aims to protect citizens' sports rights from legislation rather than promoting egalitarianism. At the same time, the government must transition from a dominant role to one of service and supervision, returning to a secondary position in the sports development process (56).

National fitness legislation should prioritize protecting citizens' sports rights by reshaping the legislative concept to focus on these rights. It should adhere to the principles of people's sovereignty and rights protection and include administrative power and obligation clauses that align with the actual needs of citizens' sports rights (57). In terms of legislative names, 66 out of the 130 effective national fitness local laws include the word “management” in their titles, emphasizing robust administrative oversight. It is recommended to reduce the frequency of the term “management” and opt for words like “service,” “protection,” and “guarantee,” as exemplified in documents such as the “Weihai City Public Sports Service Measures,” which emphasize the concept of rights protection. When it comes to regulating the administrative entity, the first step in the legislative process should be to revise the administrative leadership model to reduce the power of sports administration. It is recommended that the scope and extent of the power clause be limited when performing governmental duties, especially with regard to the administrative permission and administrative punishment powers of the sports administration department. Second, it is important to improve the service and supervision functions of administrative bodies, including strengthening the service supply responsibilities of administrative organs to citizens through their rights (58). Strengthen the surveillance of the sports market to protect sports rights and oversee the opening, supply, and maintenance of fitness facilities. Finally, it is essential to clarify the legal responsibilities of administrative organs and staff by adopting provisions that individually regulate the obligations of administrative organs. This aims at achieving the unity of power and responsibility. In terms of civil rights, regulations on citizens' sports rights should be strengthened. This will clarify the rights to sports enjoyed by citizens and establish the scope of freedom of their sports rights. This includes the freedom to exercise, enjoy public sports services, form or join autonomous sports organizations, and participate in sports activities. The state protects the freedom of citizens to participate in sports. Additionally, national fitness legislation should emphasize the rights of citizens to claim sports rights and stipulate that citizens have the right to seek the assistance of the state to realize these rights. For instance, request that the sports administrative body include fitness facilities and repair existing ones. Through the aforementioned legislative measures aimed at reconstructing the relationship between citizen sports rights and government duties, the goal is to achieve a balance of “power.” This change aims to translate the concept of legislative rights based on theory into practice, leading to profound changes (59).

### 6.3 Explore various dispute resolution mechanisms

Guaranteed sports disputes refer to conflicts that arise from the realization of citizen sports rights and are the primary issues in the development of the national fitness initiative. National fitness legislation should incorporate a diverse dispute resolution mechanism. It should include judicial litigation as the core process and administrative judgment, reconsideration, mediation, and arbitration as auxiliary processes. Consultation and reconciliation should complement these efforts, including public assistance, private assistance, and social support (60).

First, the liability insurance system for public sports facilities should be improved. National fitness legislation should mandate that government entities at or above the county level purchase liability insurance for schools that offer fitness facilities to the public. Builders of public sports facilities should also be required to obtain liability insurance. Considering that most public facility providers are government entities at or above the county level, the legislation should also mandate that these government entities budget for the costs of insurance purchases. Insurance must include both public liability insurance and product liability insurance (61).

Second, by enhancing the sports legal service system, educating sports law professionals, establishing a talent pool for sports legal services, and implementing other measures to improve the development of sports legal service personnel, we can achieve the dual effect of preventing and resolving sports disputes (62). Among them, judicial personnel should also strengthen their study of “Sports Law” and “National Fitness Regulations,” especially the revised “Sports Law.” Article 92, paragraph 2, and Article 98 are crucial, as they stipulate that, in addition to the Sports Arbitration Commission, the People's Court, the Arbitration Commission, and the Labor Dispute Arbitration Committee are institutions designated to resolve other sports disputes. When judges face sports-related disputes, the exclusive application of general laws often results in inconsistent verdicts in similar cases. Neglecting sports-specific regulations can lead to inadequate protection of individuals' sports-related rights (63).

## 7 Conclusions

In the context of promoting a healthy China, national fitness legislation is of great value. National fitness is not only an essential component of building a healthy China but also a strategic goal in this endeavor, which imposes greater demands on improving national fitness legislation. By examining the historical evolution of national fitness legislation, this paper argues that national fitness legislation in China is undergoing a phase of high-quality development. However, as the primary common factor in ensuring legal protection of sports rights for various groups, national fitness legislation also faces numerous challenges, such as inadequate protection of citizens' sports rights. The absence of incentive effects from the reward clause and the delayed legislation in the field of smart sports have impeded the realization of citizens' sports rights. Based on the analysis of the national fitness legislation in China, this study suggests that national fitness should be integrated with national health, highlighting the promotion of science and technology alongside ethical considerations. Furthermore, this study presents valuable recommendations on these topics to facilitate the development of national fitness legislation in China.
